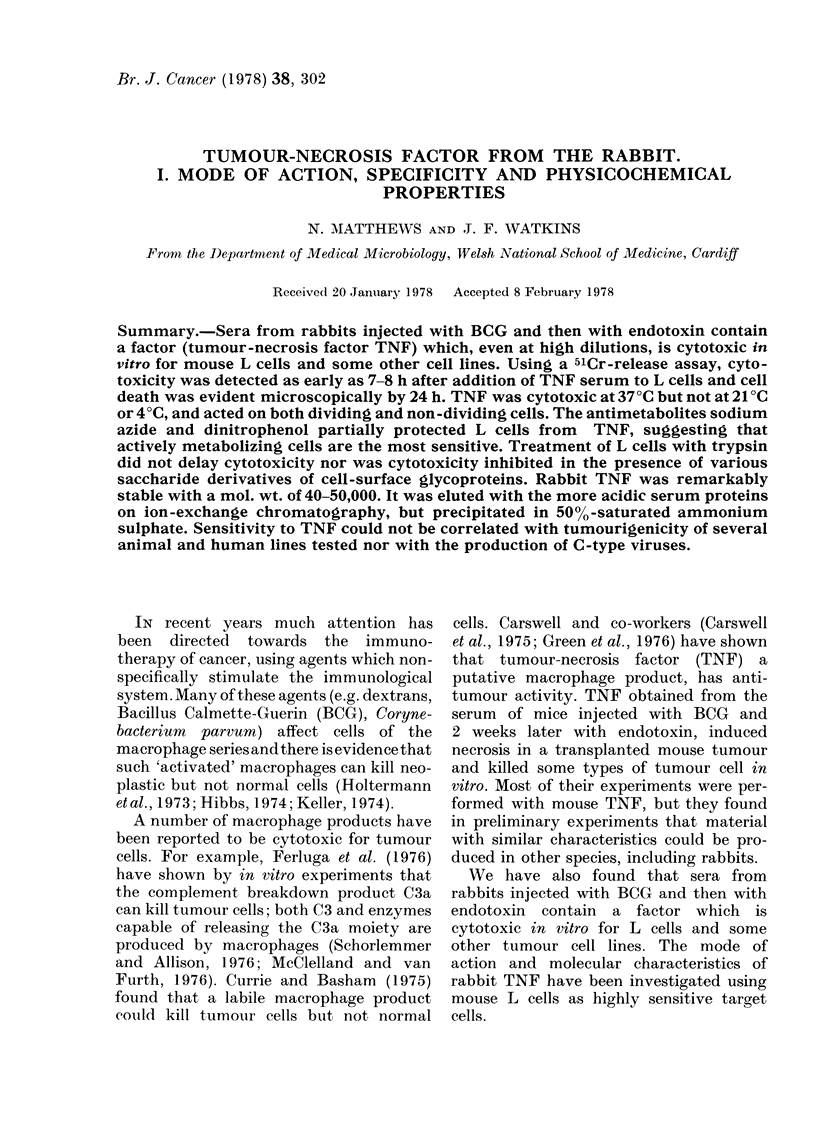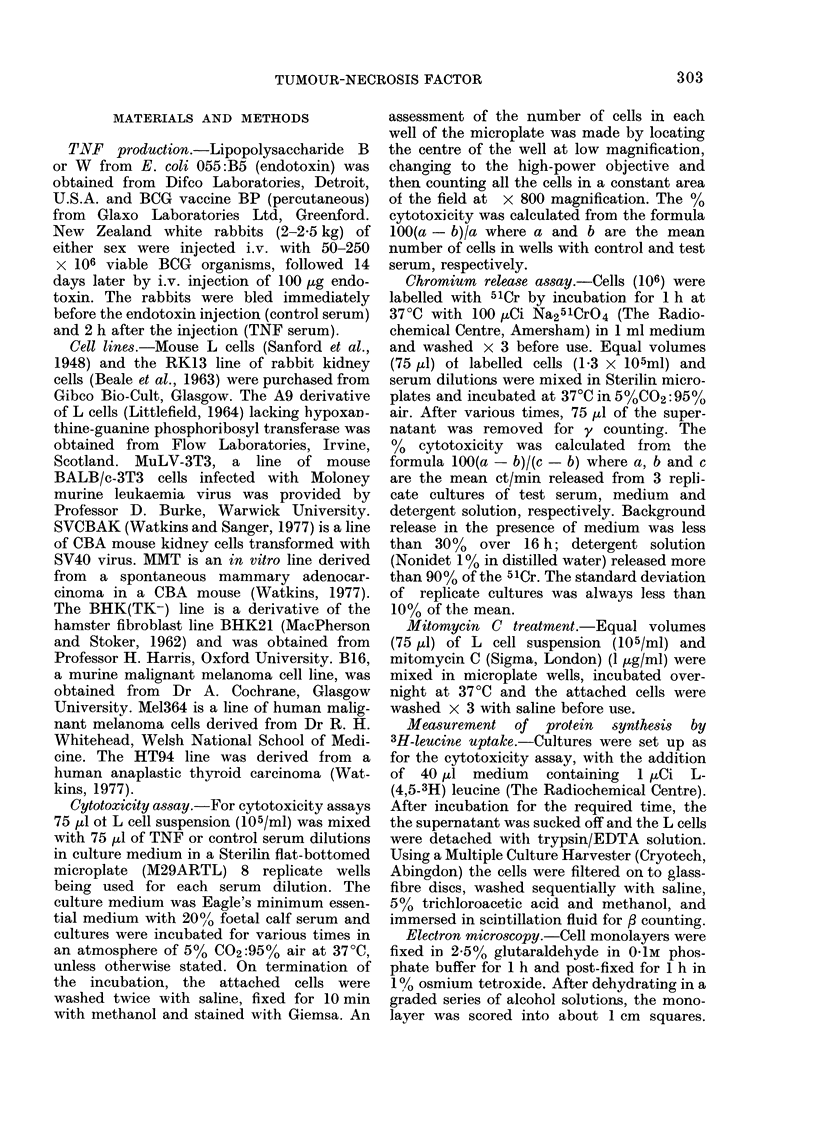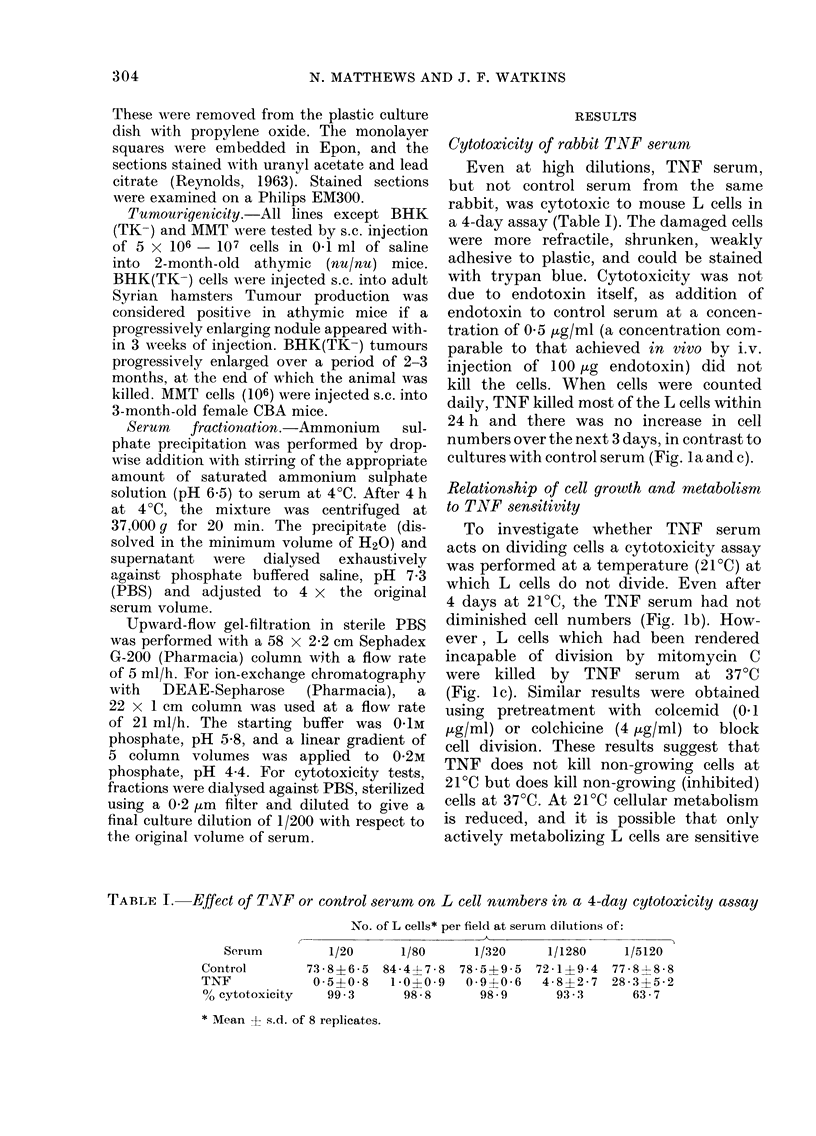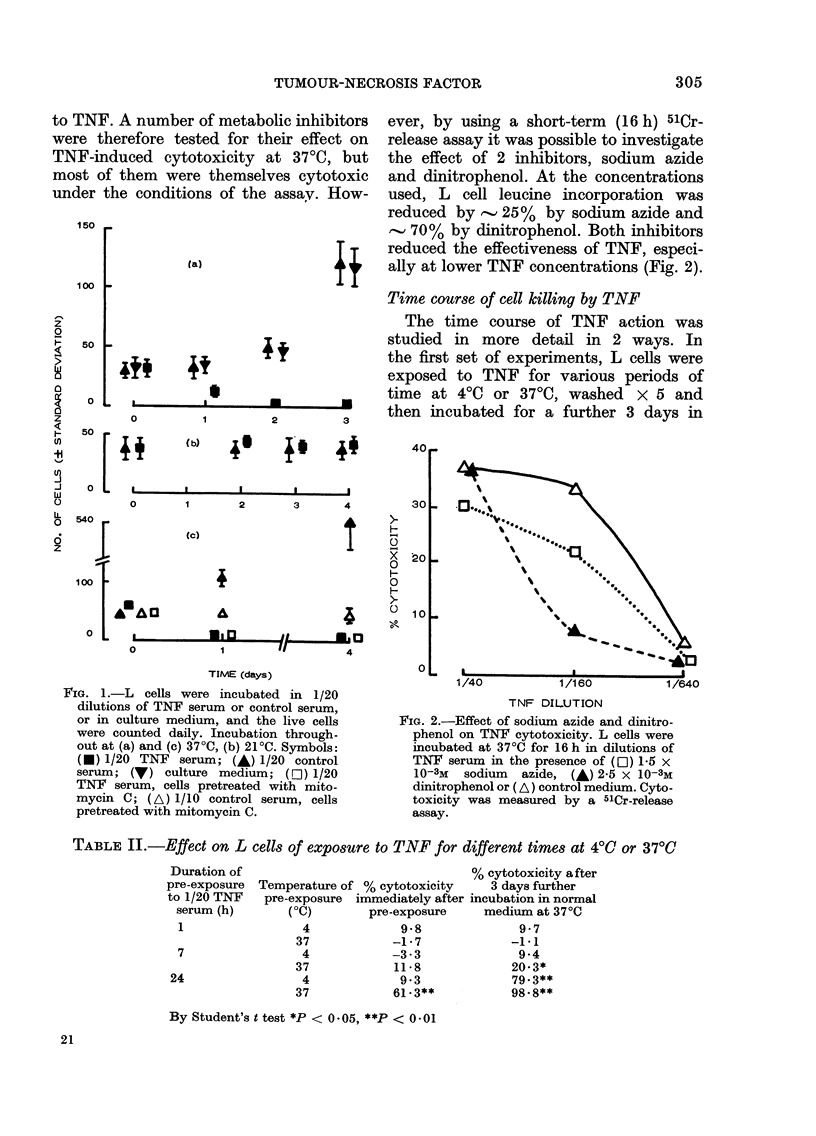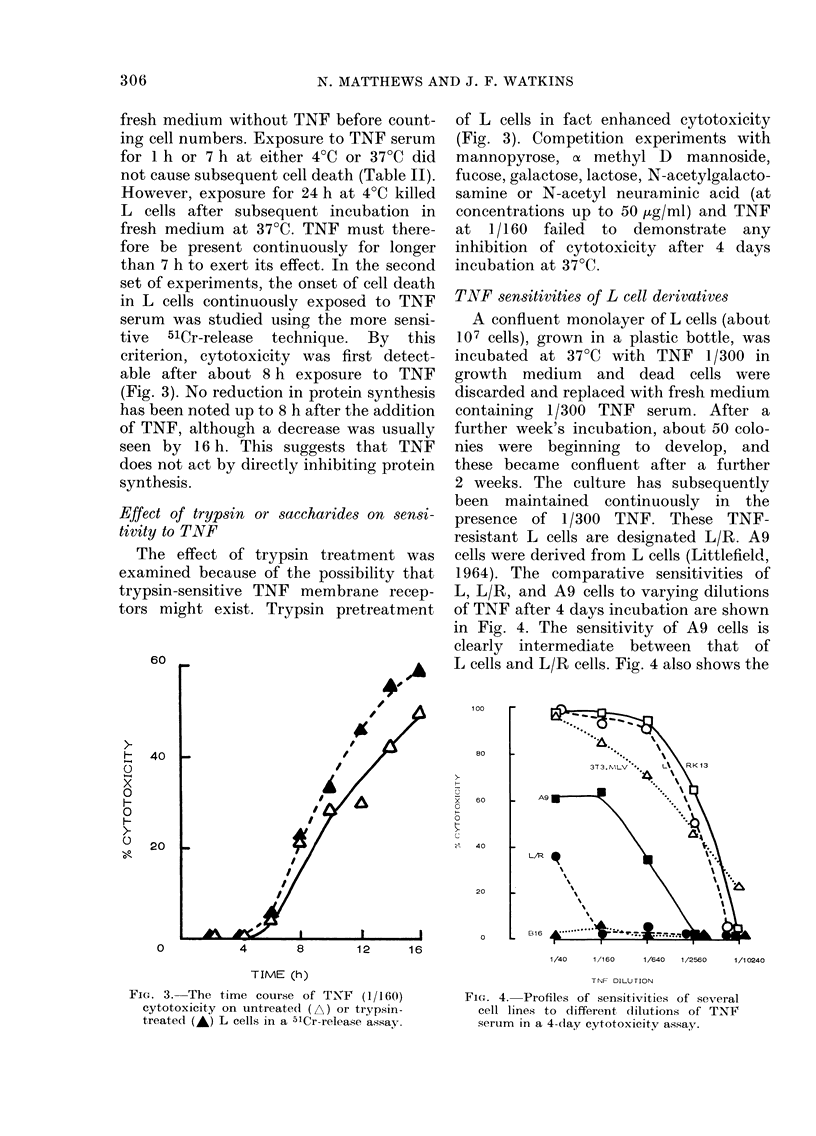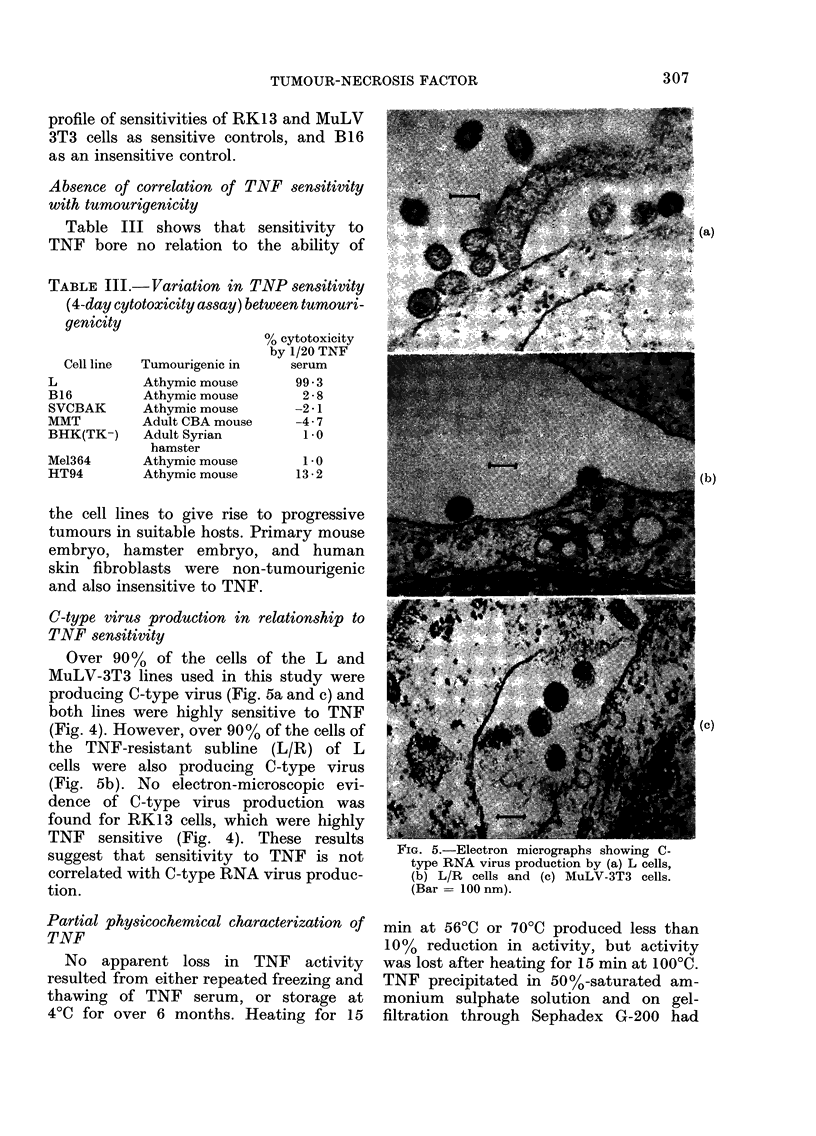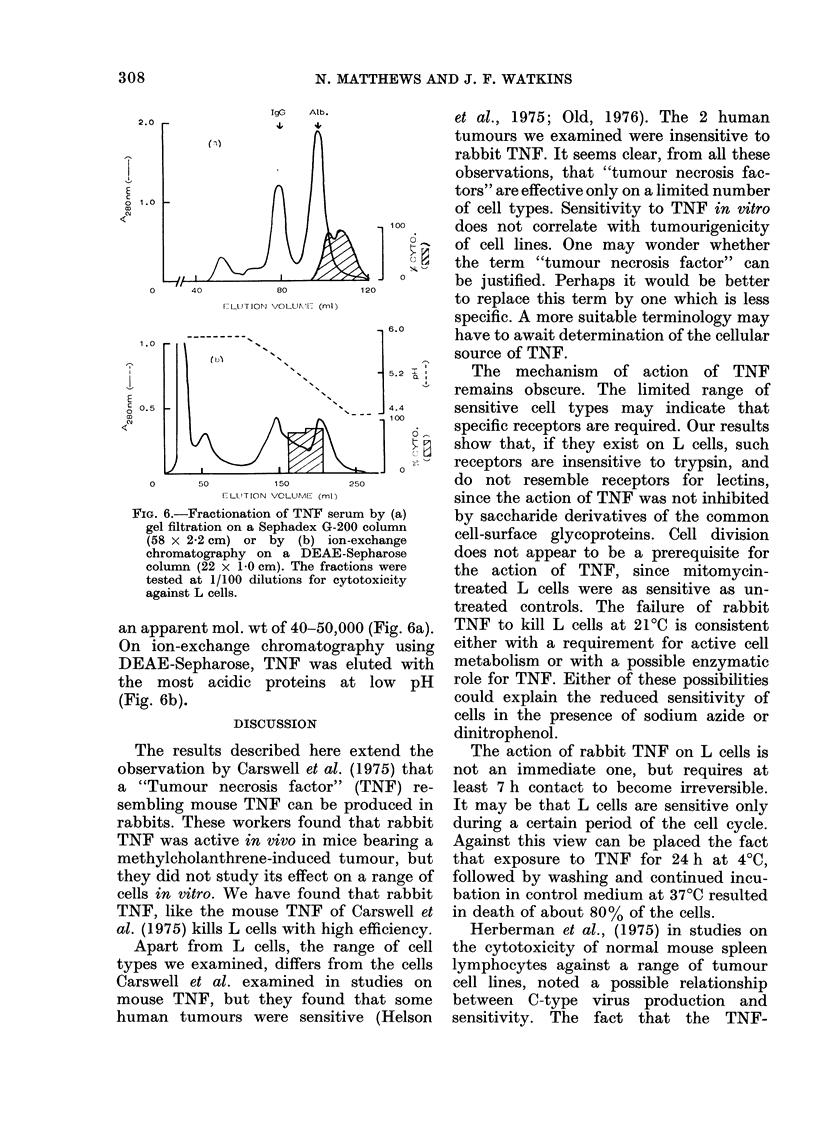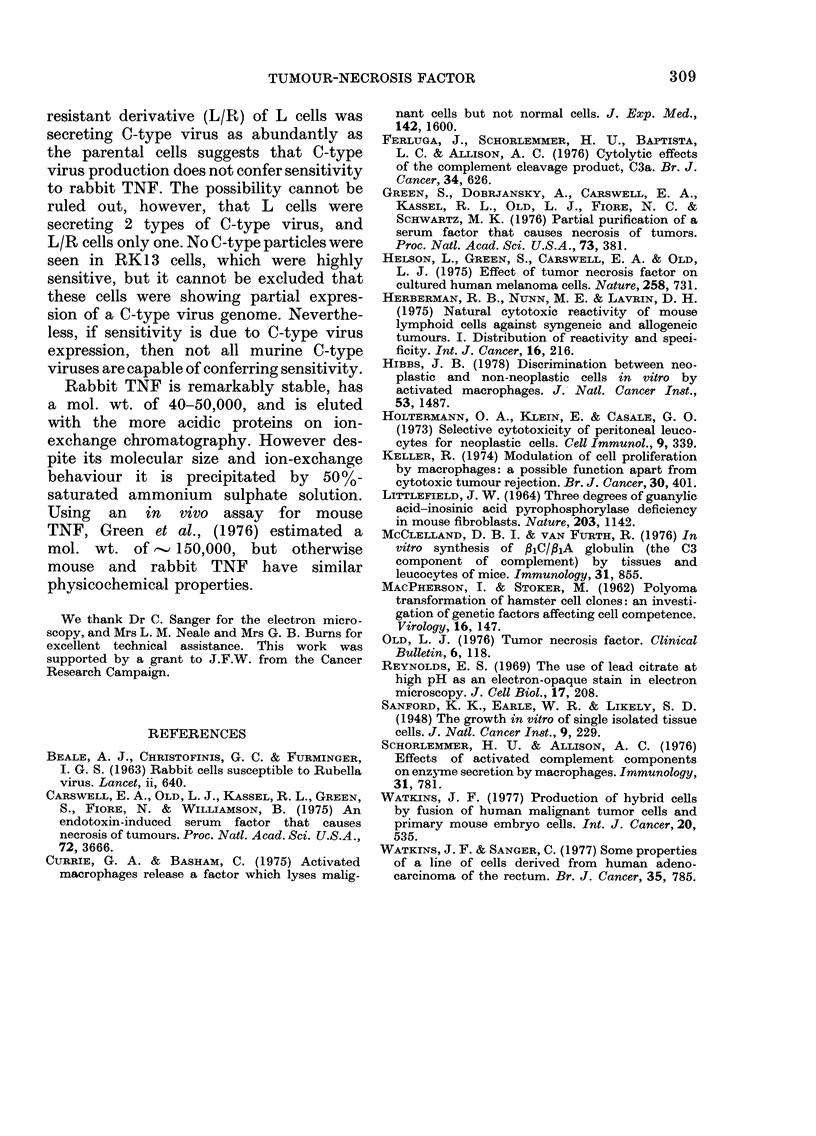# Tumour-necrosis factor from the rabbit. I. Mode of action, specificity and physicochemical properties.

**DOI:** 10.1038/bjc.1978.202

**Published:** 1978-08

**Authors:** N. Matthews, J. F. Watkins

## Abstract

**Images:**


*Br. J. Cancer* (1978) **38**, 302

### TUMOUR-NECROSIS FACTOR FROM THE RABBIT. I. MODE OF ACTION, SPECIFICITY AND PHYSICOCHEMICAL PROPERTIES

N. MATTHEWS AND J. F. WATKINS

*From the Department of Medical Microbiology, Welsh National School of Medicine, Cardiff*

Received 20 January 1978 Accepted 8 February 1978

**Summary.—Sera from rabbits injected with BCG and then with endotoxin contain a factor (tumour-necrosis factor TNF) which, even at high dilutions, is cytotoxic *in vitro* for mouse L cells and some other cell lines. Using a $^{51}Cr$-release assay, cytotoxicity was detected as early as 7–8 h after addition of TNF serum to L cells and cell death was evident microscopically by 24 h. TNF was cytotoxic at 37 °C but not at 21 °C or 4 °C, and acted on both dividing and non-dividing cells. The antimetabolites sodium azide and dinitrophenol partially protected L cells from TNF, suggesting that actively metabolizing cells are the most sensitive. Treatment of L cells with trypsin did not delay cytotoxicity nor was cytotoxicity inhibited in the presence of various saccharide derivatives of cell-surface glycoproteins. Rabbit TNF was remarkably stable with a mol. wt. of 40–50,000. It was eluted with the more acidic serum proteins on ion-exchange chromatography, but precipitated in 50%-saturated ammonium sulphate. Sensitivity to TNF could not be correlated with tumourigenicity of several animal and human lines tested nor with the production of C-type viruses.**

In recent years much attention has been directed towards the immunotherapy of cancer, using agents which non-specifically stimulate the immunological system. Many of these agents (e.g. dextrans, Bacillus Calmette-Guerin (BCG), *Corynebacterium parvum*) affect cells of the macrophage series and there is evidence that such 'activated' macrophages can kill neoplastic but not normal cells (Holtermann *et al.*, 1973; Hibbs, 1974; Keller, 1974).

A number of macrophage products have been reported to be cytotoxic for tumour cells. For example, Ferluga *et al.* (1976) have shown by *in vitro* experiments that the complement breakdown product C3a can kill tumour cells; both C3 and enzymes capable of releasing the C3a moiety are produced by macrophages (Schorlemmer and Allison, 1976; McClelland and van Furth, 1976). Currie and Basham (1975) found that a labile macrophage product could kill tumour cells but not normal cells. Carswell and co-workers (Carswell *et al.*, 1975; Green *et al.*, 1976) have shown that tumour-necrosis factor (TNF) a putative macrophage product, has anti-tumour activity. TNF obtained from the serum of mice injected with BCG and 2 weeks later with endotoxin, induced necrosis in a transplanted mouse tumour and killed some types of tumour cell *in vitro*. Most of their experiments were performed with mouse TNF, but they found in preliminary experiments that material with similar characteristics could be produced in other species, including rabbits.

We have also found that sera from rabbits injected with BCG and then with endotoxin contain a factor which is cytotoxic *in vitro* for L cells and some other tumour cell lines. The mode of action and molecular characteristics of rabbit TNF have been investigated using mouse L cells as highly sensitive target cells.

### MATERIALS AND METHODS

*TNF production.*—Lipopolysaccharide B or W from *E. coli* 055:B5 (endotoxin) was obtained from Difco Laboratories, Detroit, U.S.A. and BCG vaccine BP (percutaneous) from Glaxo Laboratories Ltd, Greenford. New Zealand white rabbits (2–2·5 kg) of either sex were injected i.v. with 50–250 $\times 10^6$ viable BCG organisms, followed 14 days later by i.v. injection of 100 $\mu$g endotoxin. The rabbits were bled immediately before the endotoxin injection (control serum) and 2 h after the injection (TNF serum).

*Cell lines.*—Mouse L cells (Sanford *et al.*, 1948) and the RK13 line of rabbit kidney cells (Beale *et al.*, 1963) were purchased from Gibco Bio-Cult, Glasgow. The A9 derivative of L cells (Littlefield, 1964) lacking hypoxanthine-guanine phosphoribosyl transferase was obtained from Flow Laboratories, Irvine, Scotland. MuLV-3T3, a line of mouse BALB/c-3T3 cells infected with Moloney murine leukaemia virus was provided by Professor D. Burke, Warwick University. SVCBAK (Watkins and Sanger, 1977) is a line of CBA mouse kidney cells transformed with SV40 virus. MMT is an *in vitro* line derived from a spontaneous mammary adenocarcinoma in a CBA mouse (Watkins, 1977). The BHK(TK$^-$) line is a derivative of the hamster fibroblast line BHK21 (MacPherson and Stoker, 1962) and was obtained from Professor H. Harris, Oxford University. B16, a murine malignant melanoma cell line, was obtained from Dr A. Cochrane, Glasgow University. Mel364 is a line of human malignant melanoma cells derived from Dr R. H. Whitehead, Welsh National School of Medicine. The HT94 line was derived from a human anaplastic thyroid carcinoma (Watkins, 1977).

*Cytotoxicity assay.*—For cytotoxicity assays 75 $\mu$l ot L cell suspension ($10^5$/ml) was mixed with 75 $\mu$l of TNF or control serum dilutions in culture medium in a Sterilin flat-bottomed microplate (M29ARTL) 8 replicate wells being used for each serum dilution. The culture medium was Eagle's minimum essential medium with 20% foetal calf serum and cultures were incubated for various times in an atmosphere of 5% $CO_2$:95% air at 37°C, unless otherwise stated. On termination of the incubation, the attached cells were washed twice with saline, fixed for 10 min with methanol and stained with Giemsa. An assessment of the number of cells in each well of the microplate was made by locating the centre of the well at low magnification, changing to the high-power objective and then counting all the cells in a constant area of the field at $\times$ 800 magnification. The % cytotoxicity was calculated from the formula $100(a - b)/a$ where $a$ and $b$ are the mean number of cells in wells with control and test serum, respectively.

*Chromium release assay.*—Cells ($10^6$) were labelled with $^{51}Cr$ by incubation for 1 h at 37°C with 100 $\mu$Ci $Na_2{}^{51}CrO_4$ (The Radiochemical Centre, Amersham) in 1 ml medium and washed $\times$ 3 before use. Equal volumes (75 $\mu$l) of labelled cells (1·3 $\times$ $10^5$ml) and serum dilutions were mixed in Sterilin microplates and incubated at 37°C in 5%$CO_2$:95% air. After various times, 75 $\mu$l of the supernatant was removed for $\gamma$ counting. The % cytotoxicity was calculated from the formula $100(a - b)/(c - b)$ where $a$, $b$ and $c$ are the mean ct/min released from 3 replicate cultures of test serum, medium and detergent solution, respectively. Background release in the presence of medium was less than 30% over 16 h; detergent solution (Nonidet 1% in distilled water) released more than 90% of the $^{51}Cr$. The standard deviation of replicate cultures was always less than 10% of the mean.

*Mitomycin C treatment.*—Equal volumes (75 $\mu$l) of L cell suspension ($10^5$/ml) and mitomycin C (Sigma, London) (1 $\mu$g/ml) were mixed in microplate wells, incubated overnight at 37°C and the attached cells were washed $\times$ 3 with saline before use.

*Measurement of protein synthesis by $^3$H-leucine uptake.*—Cultures were set up as for the cytotoxicity assay, with the addition of 40 $\mu$l medium containing 1 $\mu$Ci L-(4,5-$^3$H) leucine (The Radiochemical Centre). After incubation for the required time, the the supernatant was sucked off and the L cells were detached with trypsin/EDTA solution. Using a Multiple Culture Harvester (Cryotech, Abingdon) the cells were filtered on to glass-fibre discs, washed sequentially with saline, 5% trichloroacetic acid and methanol, and immersed in scintillation fluid for $\beta$ counting.

*Electron microscopy.*—Cell monolayers were fixed in 2·5% glutaraldehyde in 0·1M phosphate buffer for 1 h and post-fixed for 1 h in 1% osmium tetroxide. After dehydrating in a graded series of alcohol solutions, the monolayer was scored into about 1 cm squares.

These were removed from the plastic culture dish with propylene oxide. The monolayer squares were embedded in Epon, and the sections stained with uranyl acetate and lead citrate (Reynolds, 1963). Stained sections were examined on a Philips EM300.

*Tumourigenicity.*—All lines except BHK ($TK^-$) and MMT were tested by s.c. injection of $5 \times 10^6 - 10^7$ cells in 0·1 ml of saline into 2-month-old athymic (*nu/nu*) mice. BHK($TK^-$) cells were injected s.c. into adult Syrian hamsters Tumour production was considered positive in athymic mice if a progressively enlarging nodule appeared within 3 weeks of injection. BHK($TK^-$) tumours progressively enlarged over a period of 2–3 months, at the end of which the animal was killed. MMT cells ($10^6$) were injected s.c. into 3-month-old female CBA mice.

*Serum fractionation.*—Ammonium sulphate precipitation was performed by dropwise addition with stirring of the appropriate amount of saturated ammonium sulphate solution (pH 6·5) to serum at 4°C. After 4 h at 4°C, the mixture was centrifuged at 37,000 *g* for 20 min. The precipitate (dissolved in the minimum volume of $H_2O$) and supernatant were dialysed exhaustively against phosphate buffered saline, pH 7·3 (PBS) and adjusted to 4 × the original serum volume.

Upward-flow gel-filtration in sterile PBS was performed with a 58 × 2·2 cm Sephadex G-200 (Pharmacia) column with a flow rate of 5 ml/h. For ion-exchange chromatography with DEAE-Sepharose (Pharmacia), a 22 × 1 cm column was used at a flow rate of 21 ml/h. The starting buffer was 0·1M phosphate, pH 5·8, and a linear gradient of 5 column volumes was applied to 0·2M phosphate, pH 4·4. For cytotoxicity tests, fractions were dialysed against PBS, sterilized using a 0·2 μm filter and diluted to give a final culture dilution of 1/200 with respect to the original volume of serum.

### RESULTS

### *Cytotoxicity of rabbit TNF serum*

Even at high dilutions, TNF serum, but not control serum from the same rabbit, was cytotoxic to mouse L cells in a 4-day assay (Table I). The damaged cells were more refractile, shrunken, weakly adhesive to plastic, and could be stained with trypan blue. Cytotoxicity was not due to endotoxin itself, as addition of endotoxin to control serum at a concentration of 0·5 μg/ml (a concentration comparable to that achieved *in vivo* by i.v. injection of 100 μg endotoxin) did not kill the cells. When cells were counted daily, TNF killed most of the L cells within 24 h and there was no increase in cell numbers over the next 3 days, in contrast to cultures with control serum (Fig. 1a and c).

### *Relationship of cell growth and metabolism to TNF sensitivity*

To investigate whether TNF serum acts on dividing cells a cytotoxicity assay was performed at a temperature (21°C) at which L cells do not divide. Even after 4 days at 21°C, the TNF serum had not diminished cell numbers (Fig. 1b). However , L cells which had been rendered incapable of division by mitomycin C were killed by TNF serum at 37°C (Fig. 1c). Similar results were obtained using pretreatment with colcemid (0·1 μg/ml) or colchicine (4 μg/ml) to block cell division. These results suggest that TNF does not kill non-growing cells at 21°C but does kill non-growing (inhibited) cells at 37°C. At 21°C cellular metabolism is reduced, and it is possible that only actively metabolizing L cells are sensitive

TABLE I.—*Effect of TNF or control serum on L cell numbers in a 4-day cytotoxicity assay*

| Serum | No. of L cells* per field at serum dilutions of: 1/20 | 1/80 | 1/320 | 1/1280 | 1/5120 |
|---|---|---|---|---|---|
| Control | 73·8±6·5 | 84·4±7·8 | 78·5±9·5 | 72·1±9·4 | 77·8±8·8 |
| TNF | 0·5±0·8 | 1·0±0·9 | 0·9±0·6 | 4·8±2·7 | 28·3±5·2 |
| % cytotoxicity | 99·3 | 98·8 | 98·9 | 93·3 | 63·7 |

* Mean ± s.d. of 8 replicates.

to TNF. A number of metabolic inhibitors were therefore tested for their effect on TNF-induced cytotoxicity at 37°C, but most of them were themselves cytotoxic under the conditions of the assay. However, by using a short-term (16 h) $^{51}Cr$-release assay it was possible to investigate the effect of 2 inhibitors, sodium azide and dinitrophenol. At the concentrations used, L cell leucine incorporation was reduced by ~25% by sodium azide and ~70% by dinitrophenol. Both inhibitors reduced the effectiveness of TNF, especially at lower TNF concentrations (Fig. 2).

FIG. 1.—L cells were incubated in 1/20 dilutions of TNF serum or control serum, or in culture medium, and the live cells were counted daily. Incubation throughout at (a) and (c) 37°C, (b) 21°C. Symbols: (■) 1/20 TNF serum; (▲) 1/20 control serum; (▼) culture medium; (□) 1/20 TNF serum, cells pretreated with mitomycin C; (△) 1/10 control serum, cells pretreated with mitomycin C.

### *Time course of cell killing by TNF*

The time course of TNF action was studied in more detail in 2 ways. In the first set of experiments, L cells were exposed to TNF for various periods of time at 4°C or 37°C, washed × 5 and then incubated for a further 3 days in

FIG. 2.—Effect of sodium azide and dinitrophenol on TNF cytotoxicity. L cells were incubated at 37°C for 16 h in dilutions of TNF serum in the presence of (□) 1·5 × $10^{-3}$M sodium azide, (▲) 2·5 × $10^{-3}$M dinitrophenol or (△) control medium. Cytotoxicity was measured by a $^{51}Cr$-release assay.

TABLE II.—*Effect on L cells of exposure to TNF for different times at 4°C or 37°C*

| Duration of pre-exposure to 1/20 TNF serum (h) | Temperature of pre-exposure (°C) | % cytotoxicity immediately after pre-exposure | % cytotoxicity after 3 days further incubation in normal medium at 37°C |
|---|---|---|---|
| 1 | 4 | 9·8 | 9·7 |
| | 37 | −1·7 | −1·1 |
| 7 | 4 | −3·3 | 9·4 |
| | 37 | 11·8 | 20·3* |
| 24 | 4 | 9·3 | 79·3** |
| | 37 | 61·3** | 98·8** |

By Student's $t$ test $*P < 0{\cdot}05$, $**P < 0{\cdot}01$

fresh medium without TNF before counting cell numbers. Exposure to TNF serum for 1 h or 7 h at either 4°C or 37°C did not cause subsequent cell death (Table II). However, exposure for 24 h at 4°C killed L cells after subsequent incubation in fresh medium at 37°C. TNF must therefore be present continuously for longer than 7 h to exert its effect. In the second set of experiments, the onset of cell death in L cells continuously exposed to TNF serum was studied using the more sensitive $^{51}$Cr-release technique. By this criterion, cytotoxicity was first detectable after about 8 h exposure to TNF (Fig. 3). No reduction in protein synthesis has been noted up to 8 h after the addition of TNF, although a decrease was usually seen by 16 h. This suggests that TNF does not act by directly inhibiting protein synthesis.

*Effect of trypsin or saccharides on sensitivity to TNF*

The effect of trypsin treatment was examined because of the possibility that trypsin-sensitive TNF membrane receptors might exist. Trypsin pretreatment of L cells in fact enhanced cytotoxicity (Fig. 3). Competition experiments with mannopyrose, α methyl D mannoside, fucose, galactose, lactose, N-acetylgalactosamine or N-acetyl neuraminic acid (at concentrations up to 50 μg/ml) and TNF at 1/160 failed to demonstrate any inhibition of cytotoxicity after 4 days incubation at 37°C.

*TNF sensitivities of L cell derivatives*

A confluent monolayer of L cells (about $10^7$ cells), grown in a plastic bottle, was incubated at 37°C with TNF 1/300 in growth medium and dead cells were discarded and replaced with fresh medium containing 1/300 TNF serum. After a further week's incubation, about 50 colonies were beginning to develop, and these became confluent after a further 2 weeks. The culture has subsequently been maintained continuously in the presence of 1/300 TNF. These TNF-resistant L cells are designated L/R. A9 cells were derived from L cells (Littlefield, 1964). The comparative sensitivities of L, L/R, and A9 cells to varying dilutions of TNF after 4 days incubation are shown in Fig. 4. The sensitivity of A9 cells is clearly intermediate between that of L cells and L/R cells. Fig. 4 also shows the

FIG. 3.—The time course of TNF (1/160) cytotoxicity on untreated (△) or trypsin-treated (▲) L cells in a $^{51}$Cr-release assay.

FIG. 4.—Profiles of sensitivities of several cell lines to different dilutions of TNF serum in a 4-day cytotoxicity assay.

profile of sensitivities of RK13 and MuLV 3T3 cells as sensitive controls, and B16 as an insensitive control.

*Absence of correlation of TNF sensitivity with tumourigenicity*

Table III shows that sensitivity to TNF bore no relation to the ability of

TABLE III.—*Variation in TNP sensitivity (4-day cytotoxicity assay) between tumourigenicity*

| Cell line | Tumourigenic in | % cytotoxicity by 1/20 TNF serum |
|---|---|---|
| L | Athymic mouse | 99·3 |
| B16 | Athymic mouse | 2·8 |
| SVCBAK | Athymic mouse | −2·1 |
| MMT | Adult CBA mouse | −4·7 |
| BHK(TK$^-$) | Adult Syrian hamster | 1·0 |
| Mel364 | Athymic mouse | 1·0 |
| HT94 | Athymic mouse | 13·2 |

the cell lines to give rise to progressive tumours in suitable hosts. Primary mouse embryo, hamster embryo, and human skin fibroblasts were non-tumourigenic and also insensitive to TNF.

*C-type virus production in relationship to TNF sensitivity*

Over 90% of the cells of the L and MuLV-3T3 lines used in this study were producing C-type virus (Fig. 5a and c) and both lines were highly sensitive to TNF (Fig. 4). However, over 90% of the cells of the TNF-resistant subline (L/R) of L cells were also producing C-type virus (Fig. 5b). No electron-microscopic evidence of C-type virus production was found for RK13 cells, which were highly TNF sensitive (Fig. 4). These results suggest that sensitivity to TNF is not correlated with C-type RNA virus production.

FIG. 5.—Electron micrographs showing C-type RNA virus production by (a) L cells, (b) L/R cells and (c) MuLV-3T3 cells. (Bar = 100 nm).

*Partial physicochemical characterization of TNF*

No apparent loss in TNF activity resulted from either repeated freezing and thawing of TNF serum, or storage at 4°C for over 6 months. Heating for 15 min at 56°C or 70°C produced less than 10% reduction in activity, but activity was lost after heating for 15 min at 100°C. TNF precipitated in 50%-saturated ammonium sulphate solution and on gel-filtration through Sephadex G-200 had

FIG. 6.—Fractionation of TNF serum by (a) gel filtration on a Sephadex G-200 column (58 × 2·2 cm) or by (b) ion-exchange chromatography on a DEAE-Sepharose column (22 × 1·0 cm). The fractions were tested at 1/100 dilutions for cytotoxicity against L cells.

an apparent mol. wt of 40–50,000 (Fig. 6a). On ion-exchange chromatography using DEAE-Sepharose, TNF was eluted with the most acidic proteins at low pH (Fig. 6b).

### DISCUSSION

The results described here extend the observation by Carswell *et al.* (1975) that a "Tumour necrosis factor" (TNF) resembling mouse TNF can be produced in rabbits. These workers found that rabbit TNF was active *in vivo* in mice bearing a methylcholanthrene-induced tumour, but they did not study its effect on a range of cells *in vitro*. We have found that rabbit TNF, like the mouse TNF of Carswell *et al.* (1975) kills L cells with high efficiency.

Apart from L cells, the range of cell types we examined, differs from the cells Carswell *et al.* examined in studies on mouse TNF, but they found that some human tumours were sensitive (Helson *et al.*, 1975; Old, 1976). The 2 human tumours we examined were insensitive to rabbit TNF. It seems clear, from all these observations, that "tumour necrosis factors" are effective only on a limited number of cell types. Sensitivity to TNF *in vitro* does not correlate with tumourigenicity of cell lines. One may wonder whether the term "tumour necrosis factor" can be justified. Perhaps it would be better to replace this term by one which is less specific. A more suitable terminology may have to await determination of the cellular source of TNF.

The mechanism of action of TNF remains obscure. The limited range of sensitive cell types may indicate that specific receptors are required. Our results show that, if they exist on L cells, such receptors are insensitive to trypsin, and do not resemble receptors for lectins, since the action of TNF was not inhibited by saccharide derivatives of the common cell-surface glycoproteins. Cell division does not appear to be a prerequisite for the action of TNF, since mitomycin-treated L cells were as sensitive as untreated controls. The failure of rabbit TNF to kill L cells at 21°C is consistent either with a requirement for active cell metabolism or with a possible enzymatic role for TNF. Either of these possibilities could explain the reduced sensitivity of cells in the presence of sodium azide or dinitrophenol.

The action of rabbit TNF on L cells is not an immediate one, but requires at least 7 h contact to become irreversible. It may be that L cells are sensitive only during a certain period of the cell cycle. Against this view can be placed the fact that exposure to TNF for 24 h at 4°C, followed by washing and continued incubation in control medium at 37°C resulted in death of about 80% of the cells.

Herberman *et al.*, (1975) in studies on the cytotoxicity of normal mouse spleen lymphocytes against a range of tumour cell lines, noted a possible relationship between C-type virus production and sensitivity. The fact that the TNF-

resistant derivative (L/R) of L cells was secreting C-type virus as abundantly as the parental cells suggests that C-type virus production does not confer sensitivity to rabbit TNF. The possibility cannot be ruled out, however, that L cells were secreting 2 types of C-type virus, and L/R cells only one. No C-type particles were seen in RK13 cells, which were highly sensitive, but it cannot be excluded that these cells were showing partial expression of a C-type virus genome. Nevertheless, if sensitivity is due to C-type virus expression, then not all murine C-type viruses are capable of conferring sensitivity.

Rabbit TNF is remarkably stable, has a mol. wt. of 40–50,000, and is eluted with the more acidic proteins on ion-exchange chromatography. However despite its molecular size and ion-exchange behaviour it is precipitated by 50%-saturated ammonium sulphate solution. Using an *in vivo* assay for mouse TNF, Green *et al.*, (1976) estimated a mol. wt. of ~150,000, but otherwise mouse and rabbit TNF have similar physicochemical properties.

We thank Dr C. Sanger for the electron microscopy, and Mrs L. M. Neale and Mrs G. B. Burns for excellent technical assistance. This work was supported by a grant to J.F.W. from the Cancer Research Campaign.

### REFERENCES

BEALE, A. J., CHRISTOFINIS, G. C. & FURMINGER, I. G. S. (1963) Rabbit cells susceptible to Rubella virus. *Lancet*, ii, 640.

CARSWELL, E. A., OLD, L. J., KASSEL, R. L., GREEN, S., FIORE, N. & WILLIAMSON, B. (1975) An endotoxin-induced serum factor that causes necrosis of tumours. *Proc. Natl. Acad. Sci. U.S.A.*, **72**, 3666.

CURRIE, G. A. & BASHAM, C. (1975) Activated macrophages release a factor which lyses malignant cells but not normal cells. *J. Exp. Med.*, **142**, 1600.

FERLUGA, J., SCHORLEMMER, H. U., BAPTISTA, L. C. & ALLISON, A. C. (1976) Cytolytic effects of the complement cleavage product, C3a. *Br. J. Cancer*, **34**, 626.

GREEN, S., DOBRJANSKY, A., CARSWELL, E. A., KASSEL, R. L., OLD, L. J., FIORE, N. C. & SCHWARTZ, M. K. (1976) Partial purification of a serum factor that causes necrosis of tumors. *Proc. Natl. Acad. Sci. U.S.A.*, **73**, 381.

HELSON, L., GREEN, S., CARSWELL, E. A. & OLD, L. J. (1975) Effect of tumor necrosis factor on cultured human melanoma cells. *Nature*, **258**, 731.

HERBERMAN, R. B., NUNN, M. E. & LAVRIN, D. H. (1975) Natural cytotoxic reactivity of mouse lymphoid cells against syngeneic and allogeneic tumours. I. Distribution of reactivity and specificity. *Int. J. Cancer*, **16**, 216.

HIBBS, J. B. (1978) Discrimination between neoplastic and non-neoplastic cells *in vitro* by activated macrophages. *J. Natl. Cancer Inst.*, **53**, 1487.

HOLTERMANN, O. A., KLEIN, E. & CASALE, G. O. (1973) Selective cytotoxicity of peritoneal leucocytes for neoplastic cells. *Cell Immunol.*, **9**, 339.

KELLER, R. (1974) Modulation of cell proliferation by macrophages: a possible function apart from cytotoxic tumour rejection. *Br. J. Cancer*, **30**, 401.

LITTLEFIELD, J. W. (1964) Three degrees of guanylic acid–inosinic acid pyrophosphorylase deficiency in mouse fibroblasts. *Nature*, **203**, 1142.

MCCLELLAND, D. B. I. & VAN FURTH, R. (1976) *In vitro* synthesis of $\beta_1C/\beta_1A$ globulin (the C3 component of complement) by tissues and leucocytes of mice. *Immunology*, **31**, 855.

MACPHERSON, I. & STOKER, M. (1962) Polyoma transformation of hamster cell clones: an investigation of genetic factors affecting cell competence. *Virology*, **16**, 147.

OLD, L. J. (1976) Tumor necrosis factor. *Clinical Bulletin*, **6**, 118.

REYNOLDS, E. S. (1969) The use of lead citrate at high pH as an electron-opaque stain in electron microscopy. *J. Cell Biol.*, **17**, 208.

SANFORD, K. K., EARLE, W. R. & LIKELY, S. D. (1948) The growth *in vitro* of single isolated tissue cells. *J. Natl. Cancer Inst.*, **9**, 229.

SCHORLEMMER, H. U. & ALLISON, A. C. (1976) Effects of activated complement components on enzyme secretion by macrophages. *Immunology*, **31**, 781.

WATKINS, J. F. (1977) Production of hybrid cells by fusion of human malignant tumor cells and primary mouse embryo cells. *Int. J. Cancer*, **20**, 535.

WATKINS, J. F. & SANGER, C. (1977) Some properties of a line of cells derived from human adenocarcinoma of the rectum. *Br. J. Cancer*, **35**, 785.